# Perinatal outcomes among pregnant patients with peripartum coronavirus disease 2019 infection

**DOI:** 10.1007/s00404-024-07536-9

**Published:** 2024-05-06

**Authors:** Alla Saban, Noa Leybovitz Haleluya, Yael Geva, Neta Geva, Reli Hershkovitz

**Affiliations:** 1 Department of Obstetrics and Gynecology, Soroka University Medical Center, PO Box 151, 84101 Beer Sheva, Israel; 2 Faculty of Health Sciences, Ben-Gurion University of the Negev, Beer Sheva, Israel; 3 Neonatal Department, Soroka University Medical Center, Beer Sheva, Israel

**Keywords:** COVID-19, Peripartum, Perinatal outcomes, Preterm delivery

## Abstract

**Purpose:**

Evaluate maternal and neonatal outcomes in peripartum coronavirus disease 2019 (COVID-19) positive women.

**Methods:**

A retrospective cohort study was conducted, comparing outcomes between women with and without peripartum COVID-19. All singleton deliveries from June 2020 to January 2022 were included. Univariate analysis was followed by multivariate analysis.

**Results:**

Of 26,827 singleton deliveries, 563 women had peripartum COVID-19, associated with preterm deliveries both near-term and remote from term [adjusted odds ratio (aOR) 1.6 and 2.0, respectively, *p* = 0.007 and 0.003]. Women with peripartum COVID-19 had a significantly higher rate of disseminated intravascular coagulation (DIC) (aOR 23.0, *p* < 0.001). Conversely, peripartum COVID-19 peripartum COVID-19 was negatively associated with premature rupture of membranes and prolonged maternal length of stay (aOR 0.7 and 0.5, respectively, *p* = 0.006 and <0.001).

In cesarean delivery (CDs), patients with COVID-19 had higher rate of urgent CDs (75.5 vs. 56.1%, *p* < 0.001), higher rate of regional anesthesia (74.5 vs. 64.9%, *p* = 0.049), and longer anesthesia duration (86.1 vs. 53.4 min, *p* < 0.001). CD rate due to non-reassuring fetal heart rate (NRFHR) was significantly higher in women with COVID-19 (29.6 vs. 17.4%, *p* = 0.002). Conversely, CDs rate due to history of previous single CD was significantly higher in patients without COVID-19 diagnosis (13.6 vs. 4.1%, *p* = 0.006).

Concerning neonatal outcomes, an association has been observed between COVID-19 and low one-minute APGAR score <5, as well as neonatal COVID-19 infection (aOR 61.8 and 1.7 respectively, *p* < 0.001 and *p* = 0.037).

**Conclusions:**

Peripartum COVID-19 is associated with preterm deliveries, urgent CDs and DIC, potentially aligning with the infection’s pathophysiology and coagulation alterations.

## What does this study add to the clinical work

**Table:** 

This study significantly contributes to clinical practice by revealing that peripartum COVID-19 infection is associated with increased risks of preterm deliveries, urgent cesarean deliveries and disseminated intravascular coagulation. These findings emphasize the importance of heightened vigilance and tailored management strategies for pregnant women with COVID-19, enhancing our understanding of the impact of the virus on maternal and neonatal outcomes.	

## Introduction

Since its first recognition in December 2019 and throughout outbreaks of the different virus variants, coronavirus disease 2019 (COVID-19) pandemic has influenced humanity vigorously around the globe, causing social, economic, political and health implications. By the accumulation of data in the scientific world as pandemic expanded, the evidence for its nature and medical consequences are becoming clearer.

Among other populations, the pregnant women population stood out in its unique characteristics, regarding disease progression, pathophysiology, and its influence on pregnancy outcomes.

During the last years, studies were conducted concerning the perinatal outcomes of COVID-19 infection in pregnancy, demonstrating risks for several maternal, fetal, and neonatal complications. Severe maternal disease, intensive care unit (ICU) admission [1–6], preterm delivery [7], neonatal ICU admission [8], cesarean delivery (CD) [7], and hypertensive disorders of pregnancy [9] are the most consistent results related to COVID-19 infected women.

Several pathophysiologic mechanisms have been suggested, including immunologic response, placental abnormalities and exaggerated prothrombotic changes [10], contributing to the adverse perinatal outcomes observed in affected pregnant women.

Given the increased susceptibility of pregnant women, various countries implemented universal screening protocols in accordance with their respective local policies. The most prevalent screening approach involved testing pregnant women upon their admission to hospital, some of which are in labor.

Despite advancements in understanding COVID-19, its impact on peripartum outcomes remains an evolving area of research. While the acute phase of the pandemic may have subsided, new insights continue to emerge regarding the effects of COVID-19 on the peripartum period. The emergence of variants, changes in transmission dynamics, vaccination efforts, and the ongoing occurrence of COVID-19 infections in mothers all contribute to the importance of understanding its impact on peripartum health. The aim of this study is to comprehensively evaluate the maternal and neonatal outcomes of peripartum COVID-19 infection, shedding light on the unique challenges faced by pregnant individuals during this pandemic. By gaining a deeper understanding of the impact of COVID-19 on pregnancy, we aim to provide valuable insights that can enhance healthcare practices and policies, thereby advancing the quality of care and outcomes for both mothers and their neonates.

## Methods

A single center population-based retrospective cohort study comparing perinatal outcomes, specifically in close proximity to delivery, of women with and without peripartum COVID-19 infection.The study was performed at Soroka University Medical Center (SUMC), a tertiary medical center.

All singleton deliveries from June 2020 to January 2022 were included in the analysis.

We used the institutional computerized medical records. This database consists of information recorded immediately following delivery by an obstetrician and includes perinatal assessments, maternal morbidities and maternal and fetal outcomes and complications. Records were anonymised prior to analysis. The database accuracy is ensured through a meticulous review of the data by a specialist medical secretary and a consulting obstetrician.

Starting from September 2020, universal screening for COVID-19 was carried out before hospitalization using polymerase chain reaction (PCR) tests from both oral and nasal swabs.

Peripartum COVID-19 was defined as a positive PCR test result obtained within one week prior to delivery, upon hospital admission, or up to three days after the delivery, in alignment with the evolving definitions of COVID-19 infection during the study period. Positive results included both symptomatic and asymptomatic participants. Some patients received their PCR test results before going into labor, while others received them after the completion of delivery. All women gave birth in individual rooms. For women known to be COVID-19 positive during labor, the healthcare team implemented appropriate protective measures.

Sociodemographic, maternal, obstetrical and neonatal characteristics were collected using the computerized hospital medical records.

The study received the approval of the institutional review board committee of the SUMC (# SOR-0501-20). The need for informed consent was waived as participants’ identities were kept strictly anonymous and data was retrospectively collected.

Statistical analysis was performed using the SPSS package 29 edition (SPSS Inc, Chicago, IL). Categorical variable data are presented using percentiles and statistical significance was tested using the X2 and Fisher’s exact test, as appropriate. Continuous variables are presented using means and standard deviations, with *T* test used for analysis of continuous variables with normal distribution. Logistic regression models were constructed to adjust for confounders. The logistic regression estimated the adjusted odds ratios (aOR) and 95% confidence intervals (CI).

Power calculation was performed using WinPepi program, According to previous studies, the rate of pre-eclampsia or eclampsia and preterm deliveries <37 weeks in the general population was 2.5% and 6.76% of all deliveries, respectively [6, 8]. Assuming the rate in the unexposed group is equivalent to that of the entire population and given *α* = 0.05, for a rate of 3.9% and 11.05%, the calculated power is 54% and 95% for pre-eclampsia or eclampsia and preterm deliveries <37 weeks, respectively.

## Results

The study included 26,827 deliveries. Among these, 563 women were diagnosed with COVID-19 infection, while the remaining 26,264 women had no COVID-19 diagnosis.

Baseline characteristics of the study population are outlined in Table 1. Among patients diagnosed with COVID-19, there was a significantly higher proportion of Bedouin Arab women. Other baseline characteristics were similar between groups.

**Table 1:** Baseline characteristics of women with and without COVID-19 diagnosis

Characteristics	Women with COVID-19 diagnosis *n* = 563 (%)	Women without COVID-19 diagnosis *n* = 26,264 (%)	*p*
Maternal age (years)			
18	5 (0.9)	105 (0.4)	0.181
18–34	467 (82.9)	21,702 (82.6)	
≥35	91 (16.2)	4452 (17.0)	
Ethnicity			
Jewish	90 (16.0)	9787 (37.3)	<0.001
Bedouin Arab	473 (84.0)	16,466 (62.7)	
Gravidity	137 (24.5)	6323 (24.1)	0.834
1	118 (21.1)	5300 (20.2)	0.620
>2	442 (78.9)	20,910 (79.8)	
Parity			
Primiparous (parity = 1)	137 (24.5)	6323 (24.1)	
Multiparous (parity > 1)	422 (75.5)	19,886 (75.9)	
Previous CD	69 (12.3)	3765 (14.3)	0.163
Recurrent pregnancy losses^a^	31 (5.5)	1097 (4.2)	0.114
Obesity^b^	74 (16.3)	3819 (16.8)	0.763
Pre-gestational diabetes	2 (0.4)	185 (0.7)	0.446
Chronic HTN	4 (0.7)	85 (0.3)	0.117

*Covid-19* coronavirus disease 2019, *p* *p *value, *CD* cesarean delivery, *HTN* hypertension

^a^Defined as 3 or more pregnancy loses

^b^Defined as body mass index before pregnancy ≥30. BMI missing in 3635 (13.5%)

Obstetrical characteristics and perinatal outcomes of the study population are presented in Table 2. Univariate and multivariate analyses are presented. The models were adjusted for gestational age, parity, ethnicity, mode of delivery and induction of labor. Peripartum COVID-19 was associated with an increased risk of preterm deliveries both near-term (34–36 weeks) and remote from term (before 34 weeks) with an adjusted odds ratio (aOR) of 1.6 and 2.0, respectively (*p* = 0.007 and 0.003). Peripartum COVID-19 was also found to be associated with an increased risk for disseminated intravascular coagulation (DIC) aOR of 23.0 (*p* < 0.001).

**Table 2:** Obstetrical characteristics and perinatal outcomes of women with and without COVID-19 diagnosis

Characteristics	Women with COVID-19 diagnosis *n* = 563 (%)	Women without COVID-19 diagnosis *n* = 26,264 (%)	Crude OR (95% CI)	*p*	Adjusted OR^a^ (95% CI)	*p*
Mode of delivery						
Normal delivery	439 (78.0)	20,860 (79.4)	0.9 (0.75–1.12)	0.367	0.96 (0.78–1.17)	0.662
CD	4300 (16.4)	98 (17.4)	1.1 (0.86–1.34)	0.512	1.0 (0.81–1.28)	0.864
Breech delivery	2 (0.4)	24 (0.1)	3.9 (0.92–16.53)	0.103	3.2 (0.75–14.00)	0.117
Vacuum extraction	24 (4.3)	1080 (4.1)	1.0 (0.69–1.57)	0.859	1.0 (0.67–1.58)	0.883
Preterm delivery						
34–36.6 weeks	42 (7.5)	1240 (4.7)	1.5 (1.06–2.14)	0.002	1.6 (1.13–2.20)^b^	0.007^b^
<34 weeks	22 (3.9)	476 (1.8)	2.2 (1.43–3.41)	<0.001	2.0 (1.26–3.10)^b^	0.003^b^
Hypertensive disorders of pregnancy^c^	34 (6.0)	1077 (4.1)	1.5 (1.06–2.15)	0.022	1.4 (0.97–1.99)	0.070
DIC	4 (0.7)	8 (0.0)	23.5 (7.05–78.22)	<0.001	23.0 (6.50–80.99)	<0.001
GDM	20 (3.6)	1187 (4.5)	0.8 (0.50–1.22)	0.273	0.9 (0.56–1.40)	0.605
Polyhydramnios	1 (0.2)	228 (0.9)	0.2 (0.03–1.45)	0.111	0.2 (0.03–1.44)	0.120
Oligohydramnios	17 (3.0)	832 (3.2)	1.0 (058–1.55)	0.999	1.0 (0.61–1.63)	0.799
PPH	26 (4.6)	871 (3.3)	1.4 (0.95–2.10)	0.089	1.3 (0.84–1.88)	0.264
PROM	62 (11.0)	4168 (15.9)	0.6 (0.50–0.85)	0.002	0.7 (0.52–090)	0.006
PPROM	13 (2.3)	563 (2.1)	1.1 (0.62–1.88)	0.789	0.9 (0.46–1.63)	0.651
Induction of labor^d^	83 (14.7)	3903 (14.9)	1.0 (0.78–1.25)	0.938	1.2 (0.94–1.52)	0.143
Epidural anesthesia	176 (31.4)	10,226 (39.0)	0.7 (0.60–0.87)	<0.001	1.0 (0.82–1.21)	0.961
Maternal length of stay >4 days	49 (9.1)	3647 (13.9)	0.6 (0.46–0.83)	0.001	0.5 (0.39–0.73)	<0.001

*Covid-19* coronavirus disease 2019, *p* *p *value, *OR* odds ratio, *CI* confidence interval, *CD* cesarean delivery, *DIC* disseminated intravascular coagulation, *GDM* gestational diabetes mellitus, *PPH* postpartum hemorrhage, *PROM* premature rupture of membranes, *PPROM* preterm premature rupture of membranes

^a^Adjusted for gestational age, parity, ethnicity and mode of delivery

^b^The model was also adjusted for induction of labor

^c^Defined as composite of pregnancy induced hypertension, pre eclamptic toxemia and eclampsia

^d^Includes Oxytocin induction, extra-amniotic balloon induction and vaginal Prostaglandin induction

Conversely, peripartum COVID-19 was found to have a negative association with premature rupture of membranes (PROM) and prolonged maternal length of stay exceeding 4 days (which is the standard of care in our institution), aOR of 0.7 and 0.5 respectively (*p* = 0.006 and <0.001).

As for hypertensive disorders of pregnancy, though higher rates were demonstrated in the COVID-19 group, that association didn’t reach statistical significance after adjustment for potential confounders.

Table 3 presents CD characteristics of women with and without COVID-19 diagnosis. During the study period, a total of 4398 CDs were performed, 98 in women with COVID-19 diagnosis and 4300 in women without C0VID-19 diagnosis. As evident in Table 2, the rate of CDs was comparable between the two groups. However, patients with COVID-19 had a significantly higher rate of urgent CDs, 75.5 vs. 56.1% (*p* < 0.001), and higher rate of regional anesthesia 74.5 vs. 64.9% (*p* = 0.049). The mean surgery duration was comparable between the groups. However, the mean total anesthesia duration was significantly longer in patients with COVID-19, 86.1 vs 53.4 min (*p* < 0.001).

**Table 3:** Cesarean delivery characteristics of women with and without COVID-19 diagnosis

Characteristics	Women with COVID-19 diagnosis *n* = 98 (%)	Women without COVID-19 diagnosis *n* = 4300 (%)	*p*
Emergent surgery	74 (75.5)	2382 (56.1)	<0.001
Surgery duration min (mean ± SD)	36.6 ± 19.4	36.5 ± 16.6	0.953
Anesthesia duration min (mean ± SD)	86.1 ± 70.2	53.4 ± 19.6	<0.001
Type of anesthesia			
Regional alone	73 (74.5)	2788 (64.9)	0.049
General alone	24 (24.5)	1243 (28.9)	0.338
Regional & General	1 (1.0)	255 (5.9)	0.040
CD main indication			
NRFHR	29 (29.6)	747 (17.4)	0.002
Previous single CD	4 (4.1)	583 (13.6)	0.006
Previous multiple CDs	18 (18.4)	1005 (23.4)	0.246
Failed induction	1 (1.0)	64 (1.5)	1.000
NPL 1	3 (3.1)	158 (3.7)	1.000
NPL2	6 (6.1)	300 (7.0)	0.742
Breech presentation	7 (7.1)	464 (10.8)	0.248
Other non-vertex presentation	2 (2.0)	138 (3.2)	0.515
Cord prolapse	1 (1.0)	68 (1.6)	1.000
Placenta previa	4 (4.1)	76 (1.8)	0.102
Suspected macrosomia	1 (1.0)	172 (4.0)	0.185
Severe PET	1 (1.0)	47 (1.1)	1.000
Suspected placental abruption	2 (2.0)	35 (0.8)	0.199

*Covid-19* coronavirus disease 2019, *p* *p* value, *SD* standard deviation, *NRFHR* non-reassuring fetal heart rate, *CD* cesarean delivery, *NPL* Non progressive Labor, *PET* pre eclamptic toxemia

In women with a COVID-19 diagnosis who underwent CD, the leading indication to perform a CD was non-reassuring fetal heart rate (NRFHR). Conversely, for women without a COVID-19 diagnosis, the most prevalent indication was a history of multiple previous CDs.

The rate of CDs performed in the indication of NRFHR was significantly higher in women with COVID-19 diagnosis, 29.6 vs. 17.4% (*p* = 0.002). Conversely, the rate of CSDs performed due to previous single CD was significantly higher in patients without COVID-19 diagnosis, with percentages of 13.6 vs. 4.1% (*p* = 0.006). The rate of other indications was comparable between the groups.

Neonatal outcomes of the study population are presented in Table 4. In a multivariate analysis, controlling for gestational age, parity, ethnicity and mode of delivery, only Infant COVID-19 infection and one-minute APGAR score <5 remained significantly associated with maternal peripartum COVID-19 infection. Adjusted odd ratio of 61.8 (*p* < 0.001) and adjusted odd ratio of 1.7 (*p* = 0.037, respectively). It is noteworthy that the five-minute APGAR score <7 demonstrated significance in the univariate analysis but did not maintain significance in the multivariate analysis.

**Table 4:** Neonatal outcomes of women with and without COVID-19 diagnosis

Characteristics	Women with COVID-19 diagnosis *n* = 563 (%)	Women without COVID-19 diagnosis *n* = 26,264 (%)	Crude OR (95% CI)	*p*	Adjusted OR^a^ (95% CI)	*p*
Infant COVID-19 infection	6 (1.1)	4 (0.0)	70.7 (19.90–251.30)	<0.001	61.8 (16.78–228.22)	<0.001
Birthweight						
2500 g (LBW)	57 (10.1)	1851 (7.0)	1.5 (1.13–1.96)	0.005	0.9 (0.60–1.28)	0.494
2500–4000 g	482 (85.6)	23,188 (88.3)	0.8 (0.62–1.00)	0.051	1.0 (0.77–1.30)	0.986
4000 g	24 (4.3)	1225 (4.7)	0.9 (0.60–1.38)	0.655	1.0 (0.64–1.50)	0.935
Neonatal 1-min APGAR score <5	22 (3.9)	481 (1.8)	2.2 (1.40–3.35)	<0.001	1.7 (1.03–2.79)	0.037
Neonatal 5-min APGAR score <7	16 (2.9)	348 (1.3)	2.2 (1.30–3.61)	0.002	1.67 (0.92–3.01)	0.090
NICU hospitalization	20 (3.6)	667 (2.5)	1.4 (0.90–2.22)	0.132	0.9 (0.54–1.60)	0.805
Hypothermia	2 (0.4)	28 (0.1)	3.3 (0.79–14.06)	0.081	2.8 (0.66–12.00)	0.160
Hypoglycemia	7 (1.3)	187 (0.7)	1.8 (0.83–3.77)	0.130	1.39 (0.63–3.08)	0.412
Hypocalcemia	1 (0.2)	19 (0.1)	2.5 (0.33–18.39)	0.346	1.6 (0.20–12.15)	0.667
Meconium aspiration syndrome	1 (0.2)	23 (0.1)	2.3 (0.27–15.06)	0.399	2.2 (0.30–16.79)	0.432
Sepsis	0 (0.0)	27 (0.1)	–	1.000	–	0.992
Hyperbilirubinemia requiring treatment	38 (6.7)	2122 (8.1)	0.8 (0.59–1.15)	0.250	0.8 (0.55–1.10)	0.152
RDS	13 (2.3)	310 (1.2)	1.98 (1.1–3.5)	0.015	1.5 (0.79–2.93)	0.207
NEC	0 (0.0)	23 (0.1)	–	1.000	–	0.991
IVH	1 (0.2)	29 (0.1)	1.6 (0.22–11.90)	0.469	0.7 (0.05–7.05)	0.662
Pulmonary HTN	2 (0.4)	65 (0.2)	1.44 (0.35–5.88)	0.653	1.1 (0.27–4.69)	0.861
HIE	1 (0.2)	39 (0.1)	1.2 (1.16–8.72)	0.572	1.1 (0.15–8.25)	0.907
Perinatal mortality	9 (1.6)	271 (1.0)	1.6 (0.80–3.04)	0.190	1.1 (0.51–2.53)	0.766
APD or IPD	7 (1.2)	176 (0.7)	1.9 (0.87–3.99)	0.102	1.5 (0.63–3.57)	0.365
Neonatal mortality	2 (0.4)	95 (0.4)	1.0 (0.24–3.99)	1.000	0.7 (0.15–3.01)	0.607
Infant length of stay >4 days	91 (16.4)	3975 (15.3)	1.1 (0.87–1.36)	0.474	0.9 (0.70–1.16)	0.422
Composite of adverse neonatal outcomes^b^	109 (19.4)	4848 (18.5)	1.1 (0.86–1.31)	0.585	0.9 (0.69–1.10)	0.241

*Covid-19* coronavirus disease 2019, *p* *p*-value

*OR* odds ratio, *CI* confidence interval, *LBW* low birth weight, *NICU* neonatal intensive care unit, *RDS* respiratory distress syndrome, *NEC* necrotizing enterocolitis, *IVH* intraventricular hemorrhage,Pulmonary HTN Pulmonary hypertension, *HIE* Hypoxic ischemic encephalopathy, *APD* antepartum death and *IPD* intrapartum death

^a^Adjusted for gestational age, parity, ethnicity and mode of delivery

^b^Defined as composite of 1-minute APGAR score <5,5-minute APGAR score <7, NICU hospitalization, hypothermia, hypoglycemia, hypocalcemia, meconium aspiration syndrome,sepsis, hyperbilirubinemia requiring treatment, RDS, NEC, IVH , pulmonary HTN, HIE, Perinatal mortality and neonatal length of stay > 4 days

## Discussion

In this large retrospective cohort study, we found an increased risk for obstetrical and neonatal complications in pregnant women with COVID-19 diagnosis. Specifically, an increased risk of preterm deliveries and DIC was noted. These associations remained significant after controlling for potentially important confounders.

Preterm deliveries are a leading cause of infant morbidity and mortality worldwide [11, 12]. One of the well-established etiologic mechanisms associated with preterm deliveries is an increased inflammatory response. Activated neutrophils and macrophages and proinflammatory mediators [eg, interleukin (IL) 1, 6, and 8; tumor necrosis factor (TNF) and matrix metalloproteinases (MMPs)] were demonstrated in the amniotic fluid of women with preterm labor [13]. These mediators enhance prostaglandin production by inducing cyclooxygenase 2 expression in the amnion and decidua [14, 15] and enhance the expression of various MMPs in the cervix, which degrade the extracellular matrix and lead to cervical dynamics [16–18]. Wei et al. demonstrated elevated cervicovaginal and amniotic fluid IL-6 levels at midgestation in women with preterm delivery [19].

One explanation to the higher rates of preterm delivery in women infected by COVID-19 is that the activation of pro-inflammatory mediators found in preterm delivery pathogenesis may share common pathways also in COVID -19 infection, such as macrophages or IL-6 [20, 21]. There is evidence that circulating IL-6 levels are closely linked to the severity of COVID -19 infections [22]. Another explanation for this higher risk is induced pre-term deliveries indicated by maternal conditions, such as severe pre-eclampsia and sepsis.

DIC is a well-known complication of COVID-19 disease and has been reported in severely affected patients. Elevated levels of D-dimer have been observed to correlate with illness severity; D-dimer is a degradation product of cross-linked fibrin indicating augmented thrombin generation and fibrin dissolution by plasmin [23]. COVID-19-associated coagulopathy, also termed as thrombo-inflammation was described in a number of previous studies [24, 25]. Abnormal coagulation dynamics, including DIC, pulmonary embolism (PE), venous thromboembolism (VTE) and risk of thrombosis are often associated with the severity of COVID-19. COVID-19 is also a well-known risk factor for VTE, arterial thromboses and microvascular thrombosis [26–28]. Higher plasma IL-6 and TNFα levels may trigger platelet activation and coagulation, and in turn aggravate thrombosis and hypercoagulation in severe COVID-19 patients. We suggest this mechanism may be related to our findings of a higher risk for DIC in the group of COVID-19 positive patients [29].

In the context of neonatal outcomes, after controlling for confounders, only neonatal infection with COVID-19 and low one-minute Apgar score remained significantly associated with maternal peripartum COVID-19 infection. It is noteworthy to emphasize that the incidence of neonatal infection attributed to COVID-19 was remarkably low, even among infants born to mothers with COVID-19 infection, with a rate as low as 1.1%. None of the other neonatal morbidities were found to be significantly associated with maternal COVID-19 infection, either individually or as part of a composite measure.

Our analysis demonstrated a negative association with PROM and an extended maternal length of stay. The lower rate of PROM in women with peripartum COVID-19 remains an intriguing finding. While the precise mechanisms are not fully understood, it could be influenced by a complex interplay of multiple factors. Further research is needed to elucidate these relationships. The shorter maternal length of stay could be related to women’s preferences for earlier discharge, potentially as a result of the desire to shorten the period of isolation during hospitalization.

This study has few points of strength. The main advantage relates to the large cohort size. In addition, due to the fact that SUMC is the sole tertiary hospital in the entire Negev region and the fact that this area is characterized by positive immigration, selection bias is probably minimal. Also, broad inclusion criteria and limited exclusion criteria in our study produce a study population that is representative of the target population.

However, this study has also few limitations. The main limitation lies within its retrospective design. As a population-level analysis, our study can provide evidence only of association and not of causation. Nevertheless, there seems to be a biological plausibility for the observed association. Another limitation relates to the fact that several potentially important confounders were not available for analysis, such as environmental exposures and COVID-19 severity and whether patients were symptomatic or not. These important points remain to be investigated.

## Conclusion

Our study reveals the independent association of COVID-19 infection in pregnant women with preterm delivery, DIC, increased risk for low one-minute APGAR scores and the complex nature of PROM. These findings highlight the importance of vigilant monitoring and care for pregnant women with COVID-19. Additionally, there is a compelling need for further investigations into the potential long-term consequences of infection, both for symptomatic and asymptomatic pregnant individuals and their offspring.

## Data Availability

The data analyzed during the current study are not publicly available due to data sharing policies.
